# Advances in stem cell therapy for erectile dysfunction: preclinical evidence and emerging therapeutic approaches

**DOI:** 10.3389/fmed.2025.1519095

**Published:** 2025-04-02

**Authors:** Xiaoliang Fu, Azar Sheikholeslami, Ulanbek Zhanbyrbekuly, Faezeh Davoodi Asl, Nadiar M. Mussin, Hoda Fazaeli, Karim Daniyalov, Nader Tanideh, Mahdi Mahdipour, Madina A. Kurmanalina, Amin Tamadon

**Affiliations:** ^1^Department of Urology, Xianyang Central Hospital, Xianyang, China; ^2^Department of Mesenchymal Stem Cells, Academic Center for Education, Culture, and Research (ACECR), Qom, Iran; ^3^Department of Urology and Andrology, Astana Medical University, Astana, Kazakhstan; ^4^Department of General Surgery, West Kazakhstan Marat Ospanov Medical University, Aktobe, Kazakhstan; ^5^Department of Pharmacology, Medical School, Shiraz University of Medical Sciences, Shiraz, Iran; ^6^Stem Cells Technology Research Center, Shiraz University of Medical Sciences, Shiraz, Iran; ^7^PerciaVista R&D Co., Shiraz, Iran; ^8^Stem Cell Research Center, Tabriz University of Medical Sciences, Tabriz, Iran; ^9^Department of Applied Cell Sciences, Faculty of Advanced Medical Sciences, Tabriz University of Medical Sciences, Tabriz, Iran; ^10^Department of Therapeutic and Prosthetic Dentistry, West Kazakhstan Marat Ospanov Medical University, Aktobe, Kazakhstan; ^11^Department of Natural Sciences, West-Kazakhstan Marat Ospanov Medical University, Aktobe, Kazakhstan

**Keywords:** erectile dysfunction, stem cell therapy, cardiovascular diseases, mesenchymal stem cells, phosphodiesterase inhibitors

## Abstract

The inability to get or sustain an erection strong enough for fulfilling sexual performance is the hallmark of the common disorder known as erectile dysfunction (ED). It mostly affects a significant percentage of men worldwide, particularly those aged 40 to 70. Even though phosphodiesterase type 5 inhibitors (PDEi) and other conventional therapies have demonstrated efficacy, they frequently prove insufficient for patients with underlying medical disorders such as diabetes, Peyronie’s disease, or post-prostatectomy. This review delves into the therapeutic capacity of stem cells for ED, emphasizing the latest clinical and preclinical studies that showcase their efficacy across various models. The review examines diverse sources of stem cells, including adipose-derived stem cells (ADSCs), bone marrow-derived stem cells (BMSCs), and other emerging sources such as urine-derived stem cells (UDSCs). Critical studies are highlighted, particularly those demonstrating the benefits of MSCs in ED models induced by cavernous nerve injury (CNI), diabetes, and other conditions. The review also explores the role of paracrine signaling, with a focus on factors like vascular endothelial growth factor (VEGF), and brain-derived neurotrophic factor (BDNF), which are involved in the regenerative process. Additionally, the capacity of stem cells with genetic modifications and the integration of stem cell therapy with adjunctive treatments such as platelet-rich plasma (PRP) and shock wave therapy are discussed. Overall, this review underscores significant progress in both clinical and preclinical studies on cell therapy for ED, paving the way for future clinical applications and innovative treatment strategies.

## Introduction

Erectile dysfunction (ED), as a sexual impotence, is the inability to achieve or maintain an adequate penile erection for satisfactory sexual intercourse (1). ED is almost a common disease and its prevalence raises with age. While there is no set time frame for diagnosing ED, some recommend that the condition should last for 6 months (1).

ED is an important but underappreciated cardiovascular risk factor that can be a symptom of a wide range of underlying diseases (2). Erectile dysfunction can be brought on by any illness process that affects the tunica albuginea, hormone levels, smooth muscle tissue, body endothelium, or penile arteries (3). Hyperlipidemia, hypertension, diabetes mellitus, cardiovascular illnesses, and other conditions are directly linked to this disease (3, 4). Another common mechanism in patients with this condition appears to be endothelial dysfunction (5). While organic diseases account for the great majority of ED patients, some patients—especially younger men—may have a main psychological problem (6).

Even when the underlying cause is organic, often psychological results from marital and relational issues, cultural expectations, anxiety, shame, loss of self-esteem, depression, and other factors are involved in ED (7). ED usually leads to significant emotional distress to patients and their partners, and it can also have a substantial effect on their quality of life (8). Fortunately, ED is almost always treatable (9). This review aims to critically evaluate the available research on the efficacy of stem cell therapy in the management of erectile dysfunction, exploring its potential mechanisms, clinical outcomes, and comparative effectiveness against traditional treatments.

## Etiology of ED

The cause of ED is often multifactorial. Even in cases where there are organic causes, depression, anxiety related to performance, and other sexual disorders might be significant contributing factors (10). Table 1 summarizes the multifactorial etiology of ED, highlighting key contributing factors along with relevant references.

**Table 1:** Causes and risk factors of erectile dysfunction (ED).

Category	Specific causes	References
Psychological factors	Depression, anxiety, performance-related stress, other sexual disorders	(10, 51)
Aging	Increased risk of ED with age	(52)
Cardiovascular diseases	Hypertension, atherosclerosis, coronary artery disease, endothelial dysfunction	(3, 5, 53–56)
Metabolic disorders	Diabetes mellitus, metabolic syndrome	(57)
Neurological conditions	Multiple sclerosis (MS), stroke	(58, 59)
Hormonal disorders	Hypogonadism, thyroid disorders	(60)
Trauma and injury	Pelvic fractures, spinal cord injury	(61)
Respiratory conditions	Sleep apnea, chronic obstructive pulmonary disease (COPD)	(62, 63)
Ophthalmologic conditions	Glaucoma	(64)
Urological disorders	Priapism, benign prostatic hyperplasia with lower urinary tract symptoms (BPH with LUTS)	(65, 66)
Iatrogenic factors	Transurethral resection of the prostate (TURP), radical prostatectomy, radiation therapy	(67–69)
Medications	Antidepressants, antihypertensives (diuretics, beta-blockers, sympathetic blockers), antipsychotics, opioids	(70–72)
Lifestyle factors	Smoking, cycling (perineal nerve compression)	(54, 73, 74)
Prognostic significance	ED as an early marker of cardiovascular disease, association with increased risk of cardiovascular events	(53–55, 75)

## Epidemiology of ED

Many patients in emergency departments do not receive proper medical attention, and many doctors are reluctant to ask about their patient’s sexual health. It is so difficult to find precise and trustworthy statistics regarding the actual prevalence of ED. According to the greatest data currently available, 52% of American males between the ages of 40 and 70 report having ED (11). Furthermore, it is estimated that at least 30 to 50 million men in the U.S. and at least 150 million men worldwide suffer from ED (12, 13).

Due to underreporting, cultural factors, and the lack of follow-up by many physicians on sexual health and private matters, the actual number of men with ED is likely underreported. Age and the existence of additional underlying disorders including diabetes, hypogonadism, and cardiovascular diseases are strongly correlated with the prevalence of ED (11). The Massachusetts Male Aging Study’s best available statistics show an overall prevalence of 52% and an incidence that rises with age (14). It is estimated that 322 million men worldwide will suffer from erectile dysfunction by the year 2025 (12).

## Pathophysiology of ED

To achieve a penile erection, the smooth muscle in the corpus cavernosum must relax. By filling the veins with blood and increasing blood flow into the corpora cavernosa, this mechanism lowers venous output (15). This mechanism is governed by the medial preoptic region of the hypothalamus and the paraventricular nuclei (15).

The parasympathetic nervous system sends signals to the parasympathetic nerves of the sacral plexus S2-S4, which subsequently sends signals to the penis through the cavernous nerves. The erection process is started by the cavernous nerve terminals releasing nitric oxide, a vasodilator (15). When nitric oxide enters smooth muscle, it increases the synthesis of cyclic guanosine monophosphate (cGMP) (16).

Calcium channels are closed and potassium channels are opened when cGMP activates protein kinase G. Owing to the relaxation of the smooth muscle within the corpus cavernosum brought on by the decrease in intracellular calcium, there is an increase in venous outflow and a decrease in arterial input (15). After the erection is formed, this mechanism produces a hard erection with little blood flow in or out. The process is reversed when the smooth muscle contracts again due to the breakdown of cGMP by penile phosphodiesterase (15). Pathologies affecting any of these processes can lead to erectile dysfunction (15, 17).

## Psychogenic ED and mental health

Psychogenic ED arises from psychological factors rather than physical causes. Identifying and addressing these factors is crucial for effective treatment (18). Key indicators of psychogenic ED include sudden onset, especially following significant life changes or new relationships; situational ED, where erectile difficulties occur only in specific contexts or with particular partners; morning erections, suggesting physical capability; and fluctuations in erection quality throughout the day. These signs suggest a psychological origin and warrant further evaluation.

Engaging mental health specialists can address underlying psychological issues contributing to ED, such as performance anxiety, treatment adherence, relationship dynamics, and expectation management (18). Even in the absence of overt psychological disorders, involving mental health professionals can enhance treatment outcomes. Discussing mental health referrals can be sensitive. To facilitate acceptance, consider presenting the referral as a standard part of ED assessment, emphasizing that it’s a one-time evaluation to rule out psychological factors, and comparing it to routine tests like blood pressure checks to highlight its importance in overall health.

Addressing psychogenic ED involves a comprehensive approach that includes identifying psychological factors, engaging mental health professionals, and normalizing the referral process (19). This holistic strategy enhances the likelihood of successful treatment and improves overall sexual health. Recent studies have shown that combining psychological interventions with pharmacological treatments can be more effective than medication alone. For instance, a systematic review found that combining psychological interventions with phosphodiesterase-5 inhibitors improved treatment outcomes for ED.

Accurate therapy of erectile dysfunction (ED) requires distinguishing between distinct psychological and organic causes of the condition and proving that the patient actually has ED and not another sort of sexual illness, such as premature ejaculation (20). The impulsive development of erectile dysfunction (especially if associated with a new partner or life-changing event), situational ED, morning erections, and variations in erection rigidity are all signs of psychogenic ED (21). Cases of ED with apparent psychological causes should be referred to a mental health specialist (22).

The involvement of mental health professionals can help address related issues, such as lowering performance anxiety, encouraging adherence to treatment, developing relationships, recognizing conflicts between individuals, and setting reasonable expectations for couples, even in the absence of obvious psychological disorders (21).

## Therapeutic approach for ED

The inability to obtain and sustain an adequate penile erection for satisfying sexual activity is known as erectile dysfunction or ED. ED affects 40% of males between the ages of 40 and 70, increasing with age and affecting millions of men globally (23). Table 2 effectively organizes the treatment methods, references, and key details.

**Table 2:** The methods of treatment for erectile dysfunction (ED).

Treatment method	Key information	References
Phosphodiesterase type 5 inhibitors (PDEi)	Most commonly used due to effectiveness and low contraindications. Non-responsive cases are common in diabetics and prostatectomy patients.	(76, 77)
Shockwave therapy	Non-invasive therapy that stimulates neovascularization and improves erectile function by enhancing blood flow.	(78)
Platelet-rich plasma (PRP) injections	Uses growth factors from platelets to promote tissue regeneration and repair damaged penile structures.	(78, 79)
Stem cell therapy (SCT)	Stem cells differentiate into neuronal, smooth muscle, and endothelial cells to restore tissue loss in ED-related penile structures.	(78, 80)
	Mesenchymal stromal/stem cells (MSC) are multipotent adult stem cells with self-renewal capabilities, minimal ethical concerns, and low carcinogenic risk.	(25, 43, 81)
	Repairs damaged endothelial and smooth muscle cells through differentiation and paracrine action.	(82, 83)
	MSCs secrete bioactive factors that contribute to tissue repair and regeneration rather than direct cellular differentiation.	(84)
	MSCs modulate intracellular nitric oxide and calcium concentrations, crucial mediators in the erectile process.	(85, 86)

Promising outcomes have been observed in multiple animal models through *in vitro* differentiation into these cell lineages. However, the paracrine influence that MSCs have on the surrounding tissues appears to be their main therapeutic function (24). However, the paracrine influence that MSCs have on the surrounding tissues appears to be their main therapeutic function (25).

## Stem cell therapy for ED in preclinical settings

Utilizing a rat model of neurogenic ED, Bochinski et al. (26) initially showed the possible therapeutic advantages of neural crest-derived stem cells in 2004. Several preclinical studies have been conducted since then to demonstrate the effectiveness of stem cell therapy for treating ED caused by a range of conditions, including age, diabetes mellitus, hypertriglyceridemia, cavernous nerve injury (CNI), and others (27, 28).

Hou et al. (29) evaluated the available data about the application of ADSCs in non-CNI ED rat models, taking into account factors such as tunica albuginea damage, smoking, diabetes, and radiation therapy. The meta-analysis’s findings demonstrated that using ADSC could help restore erectile function by regenerating damaged erectile tissues (29). Further studies discovered a group of substances released by these cells that have a paracrine impact and considerably improve ED in comparison to the usage of ADSC alone (29). Neurotrophic compounds such as hepatocyte growth factor, vascular endothelial growth factor, and brain-derived neurotrophic factor (BDNF) were among the factors that were found (29).

Following earlier studies in this field, Park et al. (27) conducted a meta-analysis in 2019 that examined 19 research with the goal of assessing the impact of ADSCs in rat models of ED caused by CNI. The main findings demonstrated that the ability of ADSCs to regenerate damaged tissues following changes in measured levels of nitric oxide synthase (NOS), cyclic guanosine monophosphate (cGMP), and the smooth muscle-to-collagen ratio, as well as penile hemodynamic parameters, intracavernosal pressure (ICP), and the mean ICP/mean arterial pressure (MAP) ratio, all contribute to improved erectile function with ADSC therapy (27).

He et al. (30) evaluated the effectiveness of ADSCs modified with VEGF and Smad7 in a CNI rat model. Erectile function significantly improved in rats treated with modified ADSCs (30). Zhang et al. (31) investigated the impact of hyaluronic acid vesicles-encapsulated urine-derived stem cells (UDSCs), finding that diabetic rats responded better to this treatment than to the injection of UDSCs alone (31).

## Stem cell therapy in clinical settings

Since 2010, approximately 25 interventional studies have been registered on ClinicalTrials.gov aiming to evaluate the safety and efficacy of stem cells in treating ED, of which nine studies have already been completed (Table 3). BMSCs and ADSCs are the main sources of stem cells used in medicine for human use. Nevertheless, MSCs from the placenta (PSCs) and umbilical cord (UCSCs) have also been employed. Direct intracavernosal injection has been the primary cell delivery technique used in the majority of published studies (ICI). Tolerability and overall safety effects on erectile function were the primary measured outcomes in the majority of investigations, which were mostly phase I/II clinical trials. All things considered, no significant adverse effects from ICI of MSCs have been documented (32).

**Table 3:** Interventional studies on stem cell therapy for erectile dysfunction (ED).

Stem cell source	Delivery method	Outcomes	References
Umbilical cord stem cells (UCSC)	Intracavernosal injection (ICI)	Spontaneous morning erections within a month, improved blood glucose levels	(33)
Placenta stem cells (PSC)	ICI	No adverse effects, improved penile arterial flow for 6 months	(34)
Bone marrow stem cells (BMSC)	ICI	Safe and effective, improved erectile function for up to 1 year	(35, 36)
BMSC for diabetic ED	ICI	Improved IIEF-15 and EHS scores, safe and effective	(37, 38)
Adipose-derived stem cells (ADSC)	ICI	4/6 had spontaneous erections within a month, improved IIEF-15 scores for 12 months	(44, 45)
ADSC + platelet-rich plasma (PRP)	ICI	Increased erectile function, no side effects	(46, 47)
Mesenchymal stem cell-derived exosomes (MSC-DE)	Intravenous + low-intensity shock wave therapy (LISWT)	Increased peak systolic velocity, improved IIEF index	(48)
Oral mucosa-derived stem cells	ICI	Enhanced sexual function	(49)
Stem cells from exfoliated deciduous teeth (SHED-CM)	ICI	Significant improvement in IIEF-5 scores	(50)

## UCSC and PSC therapy for ED

Bahk et al. (33) conducted the first documented human clinical trial using allogeneic UCSCs in an experimental environment to treat diabetes-related ED. ICI was carried out on seven men, ages 57 to 83, using 1.5 × 10^7^ cells. Most patients saw a return of spontaneous morning erections within a month, and these outcomes persisted for the duration of the 6-month follow-up period (33). Furthermore, 2 weeks following therapy, a decrease in blood glucose levels was noted, which could account for the possible advantages of MSCs in the treatment of diabetes (33). In this particular study, the treatment’s safety profile was not a quantifiable outcome (33). However, in a phase I clinical trial published by Levy et al. (34), every aspect of the safety profile was assessed. Eight patients with PDEi-unresponsive ED were monitored for 6 months in this study (34). Analysis was done on treatment side effects as well as hemodynamic and functional results following ICI of placenta-derived stem cells (PSCs) (34). There were no significant adverse effects noted, and the mean penile arterial flow improved. This improvement persisted for up to 6 months from the initiation of treatment (34).

## BMSC therapy for ED

In phase I/II clinical trials (35, 36), Treatment was provided for 18 participants (12 in the initial phase of the research) who had radical prostatectomy surgery. ICI was utilized in a dose-determination manner in conjunction with the BMSC treatments (35, 36). The researchers concluded that ICI in patients with vascular ED utilizing autologous BMSC is both safe and efficacious (35, 36). In this trial, increases in sexual pleasure and erectile function were more pronounced at the 6-month mark and persisted for a year (35, 36). The average follow-up period of 62.1 months confirmed the absence of any side effects from the treatment (35, 36). Nonetheless, data collected in the first year following treatment showed a minor decline in erectile function (35, 36). The authors also found that to sustain the therapeutic impact, more ICIs might eventually be required (35, 36).

Two successive clinical trials on diabetic patients have used autologous BMSC and allogeneic Wharton’s Jelly-derived stem cells (WJSC) to treat ED in the context of diabetes-derived ED (37, 38). In the first experiment, the International Index of Erectile Function (IIEF-15) and Erection Hardness Score (EHS) significantly improved when ICI utilizing BMSC was used. It was also safe and effective (38). In the second phase, the use of two consecutive ICIs using WJSC was examined for the first time in 22 diabetic patients with ED (37). During the 12-month follow-up, penile Doppler ultrasonography imaging (DPE) and the IIEF-15 and EHS questionnaires showed positive results for safety and efficacy (37).

## ADSC therapy for ED

Another researched MSC source for ED treatment is ADSC. Metabolically active cells known as ADSCs are important for immune system modulation, apoptosis inhibition, and the revascularization of injured tissue (39, 40). These cells are easily and widely accessible, and they share the same multipotent and unique self-renewal properties as BMSC (41, 42). By secreting extracellular matrix components, various cytokines, and other growth factors, such as VEGF and bFGF, which all have angiogenic and anti-apoptotic qualities, they also have a major paracrine influence (43).

In a 2015 study by Garber and Carlos (44), six patients with diabetes received injections of 1.5 × 10^7^ ADSC cells. ADSC acquired following culture without SVF separation was used in the ICI (44). Within a month, four out of six patients experienced spontaneous morning erections, and with PDEi support, they were able to engage in sexual activity for up to 12 months following ICI (44). These outcomes were in line with those reported in a different trial that demonstrated autologous ICI-ADSC as a secure and successful therapy for patients with ED after radical prostatectomy (45). No side effects were observed during the 12-month follow-up (45). Six months after therapy, the IIEF-15 scoring results dramatically improved, and at 12 months, the improvement persisted (45). Studies have also been conducted combining cultured ADSC with platelet-rich plasma (PRP) (46, 47). After several follow-ups, the results showed promise and increased erectile function without any unfavorable side effects (46, 47).

## Other sources of stem cells for treatment of ED

In a study by Zasieda (48), for 6 weeks, mesenchymal stem cell-derived exosomes (MSC-DE) were administered intravenously once a week. Additionally, a combination of MSC-DE injection and low-intensity shock wave therapy (LISWT) was administered twice a week, utilizing 3,000 shocks at a frequency of 3 Hz. Following treatment, the penis’s peak systolic velocity improved and its end-diastolic velocity was greatly reduced, as evidenced by a considerable rise in the IIEF index as compared to the intervention status (48).

Mirzaei et al. (49) employed an intracavernosal injection of 50–60 million stem cells that were isolated from the oral mucosa. This randomized controlled clinical trial’s findings demonstrated that injecting intracavernosal stem cells enhanced sexual function (49).

Finally, Koga and Horiguchi (50) desired to test the direct injection of stem cells produced from exfoliated deciduous teeth pulp (SHED-CM) to investigate the restoration of sexual function through cellular regeneration of injured vascular tissue in the corpus cavernosum. Following three SHED-CM treatment sessions, the IIEF-5 score significantly improved in 38 ED patients who had not previously received PDE5I or testosterone replacement therapy (50). Because of its safety profile and ability to restore vascular damage in the corpora cavernosa, SHED-CM therapy may be used as a future treatment option for erectile dysfunction patients (50).

Overall, interventional studies on stem cell therapy for ED have shown promising results, with observed improvements in erectile function. These advancements may be measured through hemodynamic parameters (mean penile arterial flow) or sexual function questionnaires (IIEF-15 or EHS). However, more research on stem cell therapy for ED is needed before it can be recommended as an effective and alternative treatment method for these patients.

## Conclusion

Erectile dysfunction (ED) represents a multifaceted condition with significant implications for both physical and psychological health ED, which can be described by the inability to get or sustain an adequate erection for satisfying sexual activity, affects a significant percentage of men globally, with prevalence increasing with age and the presence of various health conditions. The underlying etiology of ED is often complex, involving a combination of organic, psychological, and lifestyle factors. The substantial overlap between ED and other chronic health disorders is shown by the significant effects of neurological diseases, diabetes, and cardiovascular disease. Moreover, ED serves as a crucial early indicator of potential cardiovascular problems, reinforcing its role as a predictor of broader health concerns.

The treatment landscape for ED has evolved considerably, with phosphodiesterase type 5 inhibitors (PDEi) being the standard first-line therapy. However, resistance to these medications, particularly in cases involving diabetes or post-prostatectomy, underscores the need for alternative approaches. Recent advancements in stem cell therapy, including the use of MSCs derived from various sources, have shown promising results in preclinical and early clinical settings. These therapies offer potential benefits through direct cellular differentiation and paracrine effects, though further research is required to fully elucidate their mechanisms and optimize treatment protocols.

The epidemiological data indicates that ED is widely underreported, with many patients not receiving appropriate medical care due to cultural barriers or physician reluctance. Addressing these gaps in diagnosis and treatment is crucial for improving patient outcomes. Future research should focus on refining stem cell therapies, expanding treatment options for resistant cases, and enhancing early detection and management strategies. By integrating advances in medical science with a better understanding of ED’s multifactorial nature, we can significantly improve the quality of life for those affected by this condition.
